# First report of molecular characterization and phylogeny of *Trichuris fossor* Hall, 1916 (Nematoda: Trichuridae)

**DOI:** 10.21307/jofnem-2020-036

**Published:** 2020-04-24

**Authors:** Malorri R. Hughes, Deborah A. Duffield, Dana K. Howe, Dee R. Denver

**Affiliations:** 1 Department of Biology, Portland State University, 1719 SW 10th Ave, SRTC Rm 246, Portland, Oregon, 97201; 2 Department of Integrative Biology, Oregon State University, 3029 Cordley Hall, Corvallis, Oregon, 97331

**Keywords:** 18S rRNA, Western pocket gophers, *Thomomys*, Trichuridae, *Trichuris*, *Trichuris fossor*, Systematics, Whipworm

## Abstract

Because species of *Trichuris* are morphologically similar and ranges of host preference are variable, using molecular data to evaluate species delineations is essential for properly quantifying biodiversity of and relationships within Trichuridae. *Trichuris fossor* has been reported from *Thomomys* spp. (Rodentia: Geomyidae, ‘pocket gophers’) hosts based on morphological features alone. Partial 18S rRNA sequences for specimens identified as *T. fossor* based on morphology, along with sequences from 26 additional taxa, were used for a phylogenetic analysis. Evolutionary histories were constructed using maximum likelihood and Bayesian inference. In both analyses, the specimens fell within the *Trichuris* clade with 100% support and formed a distinct subclade with 100% support. These results confirm that *T. fossor* is a distinct species and represent the first molecular report for it. Relatedness among species within the family were well resolved in the BI tree. This study represents an initial effort to obtain a more comprehensive view of Trichuridae by including a new clade member, *T. fossor*. A better understanding of Trichuridae phylogeny could contribute to further characterization of host-associations, including species that infect livestock and humans.

Nematodes of the genus *Trichuris* (Roederer, 1761) (‘whipworms’) inhabit the ceca of a wide variety of mammalian hosts worldwide (Callejón et al., 2015; Robles et al., 2018). Approximately 80 species are described based mostly on reports that have used biometrics, morphological features, host(s) infected, and/or geography (Guardone et al., 2013; Doležalová et al., 2015; Callejón et al., 2016). These characteristics are not reliable in all instances – many species have similar attributes with overlapping ranges of measurements (Callejón et al., 2015, 2016; Vejl et al., 2017) and host preference may be more variable than previously thought (Doležalová et al., 2015). Approximately 24 species been reported from rodents, many of which exhibit comparable morphological patterns (Robles et al., 2014; Doležalová et al., 2015; Eberhardt et al., 2019).

Understanding the diversity and phylogeny of whipworms is important; false classification limits our acuity of biogeography and conceals the zoonotic potential of trichurids (Callejón et al., 2015; Doležalová et al., 2015). Certain species, e.g. *T. suis* (Schrank, 1788) and *T. trichiura* (Linnaeus, 1771), are problematic in developing countries and have vast socioeconomic impacts via human or livestock infections (nearly 1 billion human trichuriasis infections are reported globally each year) (Jex et al., 2014). Other species, such as *T. muris* (Schrank, 1788; Hall, 1916), have been gathering attention in biomedical research for potential use in immunosuppression therapy (Feliu et al., 2000). A more comprehensive understanding of relationships within this group would enable predictions about how close relatives interact with their host(s).

Relationships within Trichuridae have not been well resolved using genetic approaches; results differ depending on the gene(s) sequenced and the approach used for phylogenetic reconstructions (Callejón et al., 2015). Mitochondrial data, primarily *cox1*, have been commonly used and have allowed for high resolution of closely related lineages; however, it may be less credible to use with *Trichuris* species due to the degree of hybridization and maternal mitochondrial heredity seen in this genus (Callejón et al., 2015; Doležalová et al., 2015). Nuclear data have provided higher support for relationships than mitochondrial data (Doležalová et al., 2015). The nuclear ITS1-ITS2 genes offer markers that allow closely related species to be detected (Eberhardt et al., 2019) and ITS1-5.8S-ITS2 has been used to show relations among ruminant- and rodent-infecting species (Doležalová et al., 2015). However, the number of variants of RNA genes (including the ITS2 region) makes their utility in disentangling the phylogeny of *Trichuris* less opportune, particularly given that the amount of ploidy is unknown (Doležalová et al., 2015). The 18S rRNA gene has been used to infer the placement of trichurids within Nematoda as well as to elucidate relationships within Trichuridae and is less prone to result in unclear multiple alignments (Callejón et al., 2013, 2015; Guardone et al., 2013; Doležalová et al., 2015). To date, both nuclear and mitochondrial data have suggested that *Trichuris* may be a polyphyletic genus; species or groups within the genus, e.g. *T. trichiura* and *T. suis*, may also be polyphyletic (Doležalová et al., 2015).

*Trichuris fossor* (Hall, 1916) has been reported only from hosts belonging to the genus *Thomomys* (Wied-Neuwied 1839) (Rodentia: Geomyidae) (Todd and Lepp, 1972; Gardner, 1985; but see Falcón-Ordaz, 1993). Descriptions have been based on morphology and host preference and *Trichuris* from geomyid hosts have never been sequenced (Eberhardt et al., 2019). The aim of this study was to serve as the first molecular report for *T. fossor*. The 18S rRNA gene was sequenced for four specimens identified putatively as *T. fossor* collected from separate *Thomomys* (western pocket gophers) species hosts.

## Materials and methods

*Thomomys bulbivorus* (Richardson, 1829; Brandt, 1855) were salvaged from trappers working in Yamhill County, Oregon, in April 2018. *Thomomys botta* *e* (Eydoux and Gervais, 1836) *, T. mazama* (Merriam, 1897), and *T. talpoides* (Richardson, 1828) were collected from Curry, Jackson, and Grant County, respectively, Oregon in August 2019. Complete intestinal tracts were examined following procedures outlined by Gardner and Jasmer (1983). Collected parasites were stored in 95% ethanol. Based on morphology and previous records for the hosts, nematodes found in the ceca were tentatively identified as *T. fossor* (Chandler, 1945; Todd and Lepp, 1972; Gardner, 1985).

DNA extraction, amplification, and sequencing were performed on individuals from each host species. Before beginning isolation, nematodes were transferred to 1.5 mL microcentrifuge tubes and repeatedly rinsed with DI water (5 rinses of 1 mL dH_2_O) to remove all traces of ethanol. Nematodes were then transferred into fresh PCR tubes and mechanically homogenized before extracting with the DNeasy Blood and Tissue Kit (Qiagen) following the manufacturer’s protocols. Overlapping fragments of 18S rRNA were amplified using the primers G18S4F (5′-GCTTGTCTCAAAGATTAAGCC-3′), 136R (5′-TGATCCTTCTCGCAGGTTCACCTAC-3′), 652F (5′-GCAGCCGCGGTAATTCCAGCTC-3′), and 647R (5′-CATTCTTGGCAAATGCTTTCGC-3′) (Callejón et al., 2013). After PCR products were visualized on a 1.5% agarose gel, they were SPRI-purified (Elkin et al., 2001) and prepared for direct end sequencing. Sequencing reactions were processed by the Center for Genome Research and Biocomputing (CGRB; Oregon State University, Corvallis, OR).

Sequences were examined using MEGA v. 7.0.26 (Kumar et al., 2016). Forward and reverse sequences for individual segments were combined by alignment using MUSCLE, followed by combining the two overlapping segments. Low-quality ends were trimmed and a BLAST search against the NCBI nr database was performed. Sequence information from the BLAST match for 26 related taxa was incorporated into a phylogenetic comparison. These additional *Trichuris* spp. included in the analyses infect dogs, humans, pigs, sheep, and other rodents (murids, cricetids, and arvicolids). Sequences were aligned using MUSCLE, ends were trimmed, and 1,644 base pairs remained. The newly generated sequences were submitted to the GenBank database under accession numbers MT071351, MT071352, MT071353, and MT071354.

Phylogenetic analyses were performed in MEGA v. 7.0.26 (Kumar et al., 2016) and in BEAST2 v. 2.6.1 (Bouckaert et al., 2019). MEGA determined that Kimura 2-parameter with invariant sites and a gamma distribution was the best fit substitution model for this data based on Bayesian information criterion. An evolutionary history was inferred based on this model using the maximum likelihood (ML) method and a consensus tree was generated using 1,000 bootstrapping replicates in MEGA. The Bayesian inference (BI) analysis was prepared in BEAUti v. 2.6.0 (Bouckaert et al., 2019) and performed in BEAST2 v. 2.6.0 (Bouckaert et al., 2019). The analysis used the HKY substitution model with equal frequencies (K2P + I + G is not available in BEAST2) and ran for 1×10^7^ generations. Tracer v 1.7.1 (Rambaut et al., 2018) was used to evaluate convergence and ensure that effective sample size values for each parameter were met (all > 1,000). Tree files were combined in LogCombiner v. 2.6.0 (Bouckaert et al., 2019) and a maximum clade credibility (MCC) tree was constructed using TreeAnnotator v. 2.6.0 (Bouckaert et al., 2019) with posterior probabilities limited 50% and a burn-in percentage of 10%. FigTree v. 1.4.4 (http://tree.bio.ed.ac.uk/software/figtree/) was used to visualize the MCC tree.

## Results and discussion

**Figure 1:**
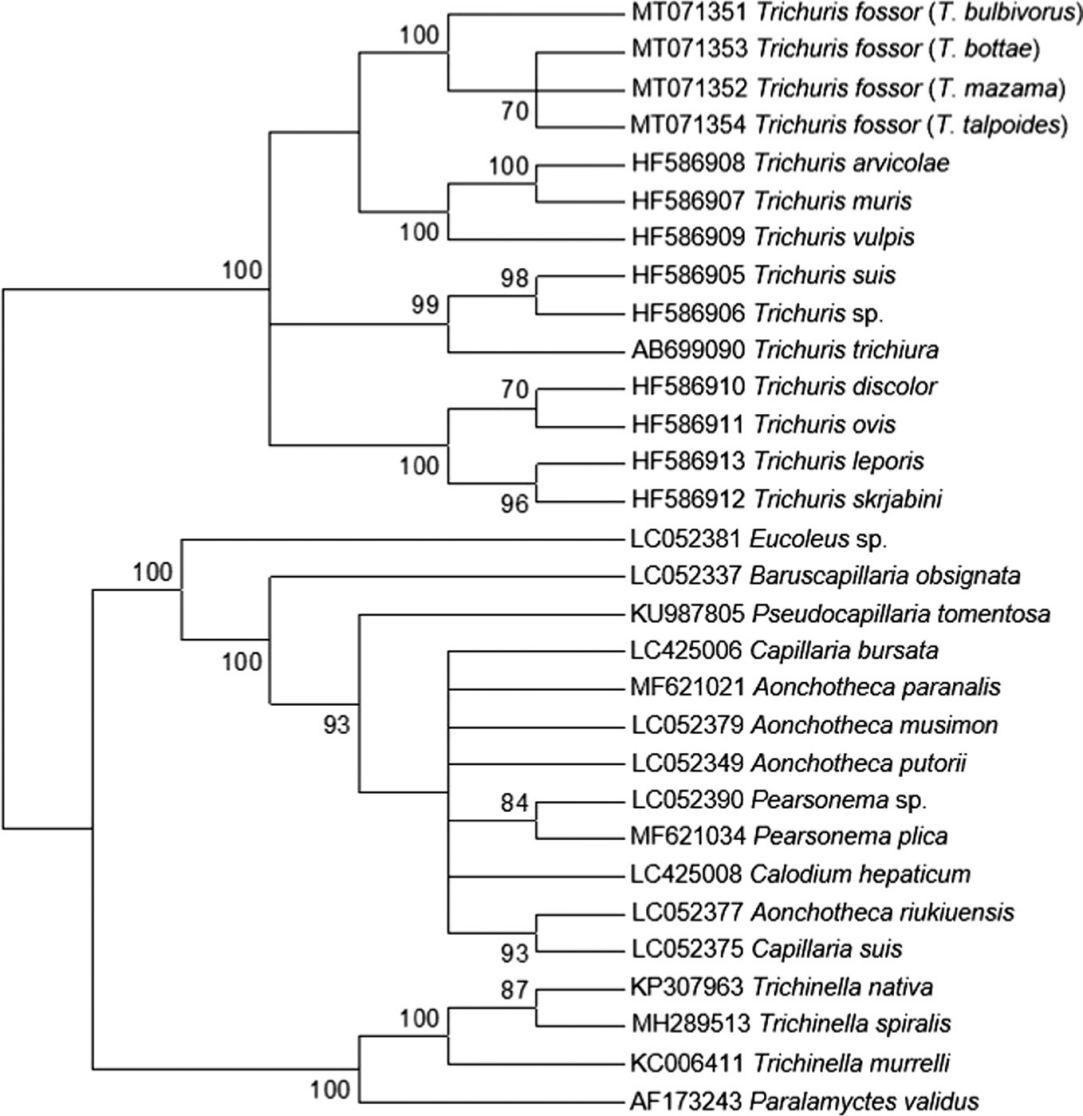
Bootstrap consensus tree (1,000 replicates) generated using the maximum likelihood method based on the K2P + I + G model. Bootstrap support values of 70% or greater are indicated next to nodes. Hosts are included in parentheses next to the *T. fossor* sequences.

**Figure 2:**
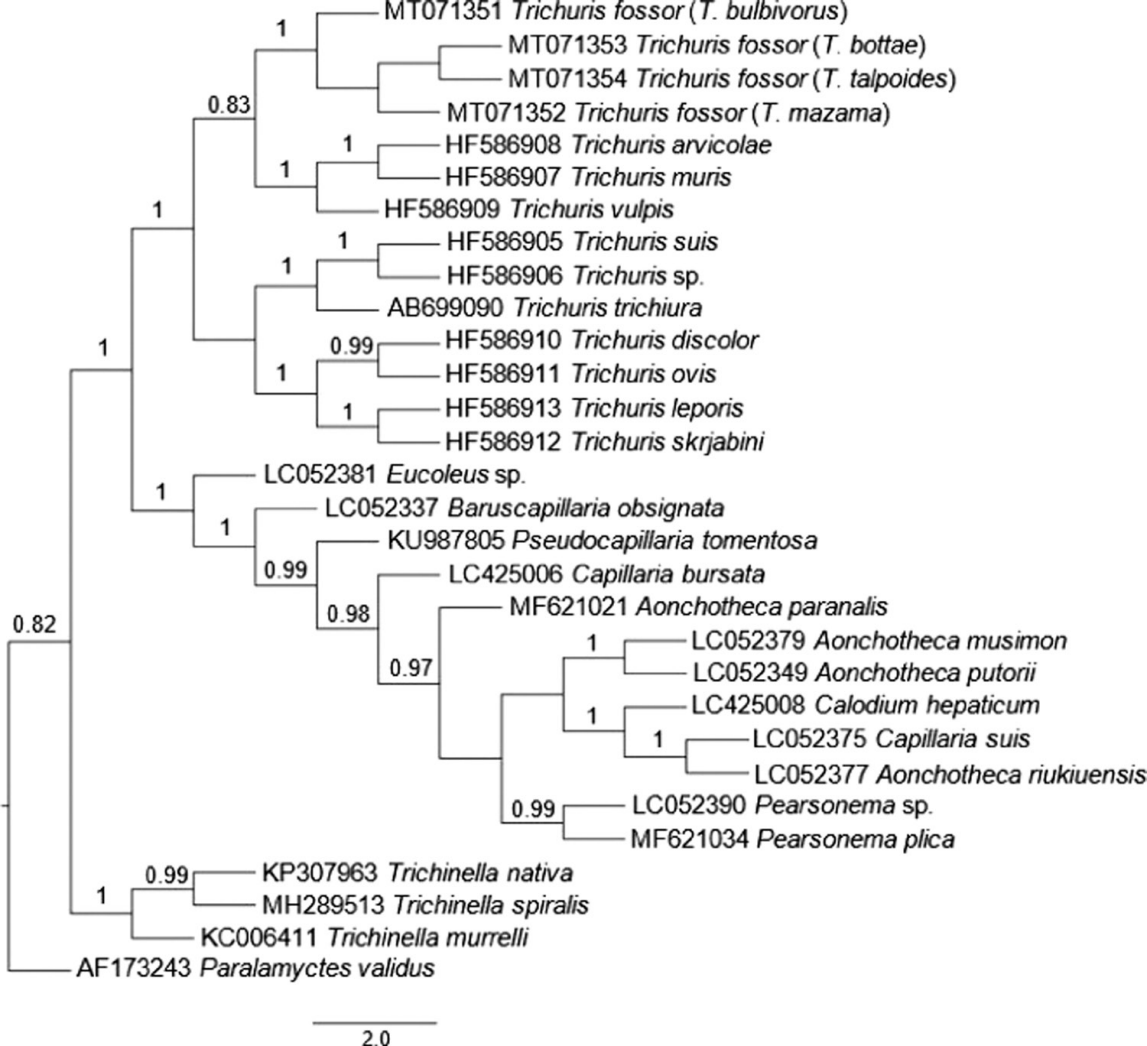
Maximum clade credibility consensus tree generated using the Bayesian inference method under the HKY model. Posterior probabilities for nodes above 70% are displayed. Hosts are included in parentheses next to the *T. fossor* sequences.

*Trichuris*, *Trichinella*, and capillariid species represented highly supported (100%) monophyletic groups (Figs. 1, 2). The capillariid and trichurid clades formed sister taxa with 100% support in both analyses. These findings are consistent with previous studies (Feldman and Ramirez, 2014; Borba et al., 2019). Our new sequences fell within the *Trichuris* clade with 100% node support and formed an independent subclade with 100% support in both analyses. Four subclades were present within *Trichuris*: (i) *T. discolor*, *T. ovis*, *T. skrjabini*, *T. leporis,* (ii) *T. trichiura*, *T. suis*, unidentified *Trichuris* sp. (= *T. colobae*, see Cutillas et al., 2014), (iii) *T. vulpis, T. muris, T. arvicolae*, and (iv) the new sequences from *T. fossor*. The composition of previously studied species in subclades 1 to 3 are consistent with results from studies that used nuclear, mitochondrial, and/or concatenated data (Callejón et al., 2013, 2015; Feldman and Ramirez, 2014; Doležalová et al., 2015). In the ML analysis, the relatedness of the *Trichuris* subclades to one another had low (< 70%) support or were unresolved (Fig. 1). The BI analysis offered better resolution among trichurids (Fig. 2). The *T. fossor* subclade was most closely related to the *T. arvicolae*, *T. muris*, and *T. vulpis* subclade; the remaining two subclades were more closely related to one another than to the other two subclades.

The results of the phylogenetic analyses verify that, based on molecular data, *T. fossor* is a distinct species. This is the first report of *T. fossor* from a *T. mazama* host*. Trichuris fossor* from *T. bulbivorus* host was an outgroup to other *T. fossor* specimens in both analyses. In the BI analysis, *T. fossor* from *T. bottae* and *T. talpoides* were sister taxa with the specimen from *T. mazama* as an outgroup. This suggests that variability likely exists among *T. fossor* from different host species, but 18S DNA is not reliable for determining whether genetic distances among *Trichuris* fall within the range of intraspecific variation (Guardone et al., 2013).

This work represents a preliminary step in investigating the phylogeny of *T. fossor*. Examining more molecular data and including different genes will likely show increased resolution of the closest relatives of *T. fossor* and provide a more comprehensive view of this phylogeny. Comparing other markers of nuclear and organellar DNA may be helpful (Doležalová et al., 2015) as well as examining mitochondrial data (e.g. *cox1* gene), especially given there is support that it is more reliable when separating closely related species than that of 18S rRNA (Guardone et al., 2013). Incorporating *T. fossor* from different host species and from different geographic areas will also be valuable as lineages within the *T. fossor* subclade could be uncovered (Callejón et al., 2010).
